# Working under short timescales to deliver a national trial: a case study of the ComFluCOV trial from a statistician’s perspective

**DOI:** 10.1186/s13063-023-07879-9

**Published:** 2024-01-23

**Authors:** Rosie Harris, Russell Thirard, Sarah Baos, Rajeka Lazarus, Rachel Todd, Jana Kirwan, Katherine Joyce, David Hutton, Maddie Clout, Heike Cappel-Porter, Lucy Culliford, Chris A. Rogers

**Affiliations:** 1 Bristol Trials Centre, University of Bristol, Bristol, UK; 2 University Hospitals Bristol and Weston NHS Foundation Trust, Bristol, UK

**Keywords:** Statistical methodology, COVID-19, Influenza, Vaccination, Randomised controlled trial

## Abstract

**Background:**

In early 2021, the Department of Health and Social Care in the UK called for research on the safety and immunogenicity of concomitant administration of COVID-19 and influenza vaccines. Co-administration of these vaccines would facilitate uptake and reduce the number of healthcare visits required. The ComFluCOV trial was designed to deliver the necessary evidence in time to inform the autumn (September–November) 2021 vaccination policy. This paper presents the statistical methodology applied to help successfully deliver the trial results in 6 months.

**Methods:**

ComFluCOV was a parallel-group multicentre randomised controlled trial managed by the Bristol Trials Centre. Two study statisticians, supported by a senior statistician, worked together on all statistical tasks. Tools were developed to aid the pre-screening process. Automated data monitoring reports of clinic data and electronic diaries were produced daily and reviewed by the trial team and feedback provided to sites. Analyses were performed independently in parallel, and derivations and results of all outcomes were compared.

**Results:**

Set-up was achieved in less than a month, and 679 participants were recruited over 8 weeks. A total of 537 [at least] daily reports outlining recruitment, protocol adherence, and data quality, and 695 daily reports of participant electronic diaries identifying any missed diary entries and adverse events were produced over a period of 16 weeks. A preliminary primary outcome analysis of validated data was reported to the Department of Health and Social Care in May 2021. The database was locked 6 weeks after the final participant follow-up and final analyses completed 3 weeks later. A pre-print publication was submitted within 14 days of the results being made available. The results were reported 6 months after first discussions about the trial.

**Conclusion:**

The statistical methodologies implemented in ComFluCOV helped to deliver the study in the timescale set. Working in a new clinical area to tight timescales was challenging. Having two statisticians working together on the study provided a quality assurance process that enabled analyses to be completed efficiently and ensured data were interpreted correctly. Processes developed could be applied to other studies to maximise quality, reduce the risk of errors, and overall provide enhanced validation methods.

**Trial registration:**

ISRCTN14391248, registered on 30 March 2021

## Background

In 2021, a vaccination strategy to manage the continuing need for mass COVID-19 vaccination and predicted higher rates of seasonal influenza was urgently needed. There were concerns that the emergence of variants and the seasonal respiratory infections could increase over the winter period. Timing of COVID-19 booster doses was likely to coincide with the influenza vaccine season. Concomitant vaccination of COVID-19 and influenza vaccines could facilitate uptake of both vaccines and reduce the number of healthcare visits, but data on safety and immunogenicity of concomitant vaccination were lacking. The UK Department of Health and Social Care (DHSC) commissioned a call for research in February 2021 to provide evidence to determine the safety and immune responses of co-administration of COVID-19 and influenza vaccines to inform the autumn (September–November) 2021 policy.

The results of the research were required by September 2021 with a preliminary safety report due in May 2021, just 3 months after trial inception. The purpose of this paper is to report the key statistical methodology which was implemented to contribute to the successful delivery of the ComFluCOV trial results to inform vaccination policy.

## Methods

### Study design

The study design and main results have been reported previously [1]. In brief, ComFluCOV was a parallel-group, placebo-controlled, multicentre randomised controlled trial (RCT) with blinding to evaluate the safety and immune response of co-administration of COVID-19 and influenza vaccines. The study was initially designed to assess two COVID-19 vaccines (ChAdOx1; Oxford–AstraZeneca and BNT162b2; Pfizer–BioNTech) and two influenza vaccines (Flucelvax cellular quadrivalent vaccine and FluAd MF59C adjuvanted trivalent vaccine [Seqirus UK; Maidenhead, UK]). A further influenza vaccine (Flublok recombinant quadrivalent vaccine (Sanofi; Paris, France)) was added shortly after the study started as requested by the DHSC, resulting in six COVID-19/influenza vaccine cohorts. Participants were randomised in a 1:1 ratio to coadministration of their second COVID-19 dose and either a seasonal influenza vaccine or a placebo. Participants, laboratory staff, and clinicians assessing causality of adverse events were blinded to treatment allocation until the trial was reported. Participants who received placebo at their randomisation visit (visit 1) received influenza at day 21 (visit 2) and vice versa. Participants were followed up to 8 weeks post-randomisation (third and last visit at day 42).

### Sample size

Initially, the target sample size was set at 504 participants (126 per COVID-19/influenza vaccine cohort) which provided 80% power (at a 2.5% one-sided significance level) to test the hypothesis that concomitant administration of COVID-19 and influenza vaccines was non-inferior to the administration of the COVID-19 vaccine alone for each COVID-19/influenza vaccine combination. Combinations of different event frequencies (reported in other COVID-19 research [2, 3]), non-inferiority margins, and power were explored to provide a sample size that was both acceptable to the clinical members of the study team and pragmatic given the accelerated timescale of the trial. A 2.5% one-sided significance level was chosen as recommended in regulatory settings [4]. The sample size did not account for dropout or for clustering which we assumed to be negligible as the intervention was standardised across centres and there was no active clinician-based intervention beyond appropriate follow-up of adverse events. The frequency of the primary outcome was assumed to be 50% in the COVID-19 alone group and an absolute increase of less than 25% of any solicited systemic events was considered non-inferior. When the trial was adapted to add a third influenza vaccine, the sample size was increased to 756 participants (504 + 2 × 126).

### Statistics team

The ComFluCOV trial was run by the Bristol Trials Centre (BTC), and a central team of statisticians, trial managers, and database managers was established to help deliver the trial, with leadership provided by a senior statistician/BTC lead for the trial. A novel approach was used to deliver the data and analyses in time for the upcoming influenza season; two trial statisticians, instead of one, with support and guidance from a senior statistician, were jointly responsible for all statistical tasks. The two trial statisticians were part of a larger statistical team at the BTC who all work closely together with similar ways of working. Considering the different stages of ComFluCOV and timelines for each stage, a risk proportionate approach was used to determine the most appropriate statistical process for each element of the trial (i.e. setup, monitoring, analysis and reporting).

### Trial set up

**Fig. 1:**
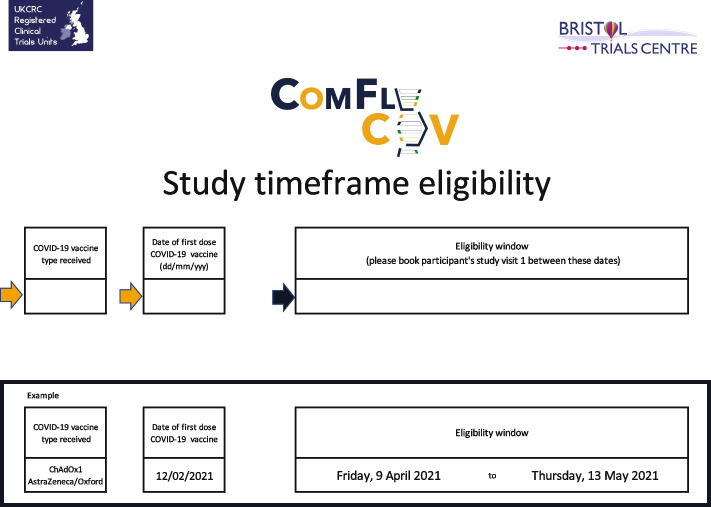
Screenshot of tool created to calculate eligibility windows

**Fig. 2:**
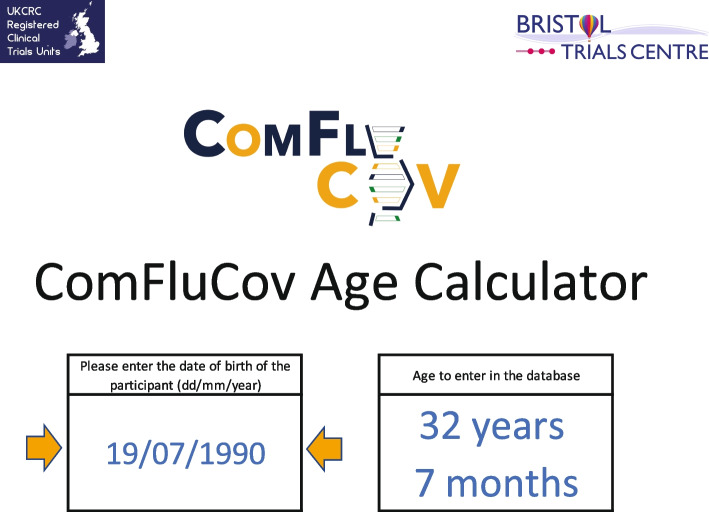
Screenshot of tool created to calculate age in years and months for entry onto database

During set-up, two bespoke tools were developed in Microsoft Excel to help sites to streamline the pre-screening process. One tool provided the time period (date range) when a volunteer was eligible to receive their second COVID-19 vaccination as part of the ComFluCOV trial based on the date of the volunteer’s first COVID-19 vaccination (Fig. 1); this helped site staff booking volunteers in for clinics to assess the eligibility of potential participants for the trial. The other tool calculated the volunteer’s age in years and months for entry onto the trial database (storage of the date of birth was not permitted by the Research Ethics Committee), to save the site staff time and increase accuracy of calculations for inclusion (Fig. 2).

The randomisation scheme was prepared by an independent statistician from the BTC who was not otherwise involved in the trial. Randomisation was blocked (using variable block sizes of 2, 4, and 6) and stratified by centre, age group (< 65 years or ≥ 65 years), and first COVID-19 vaccine received (BNT162b2 or ChAdOx1). A section of the database from another trial (ComCOV), run by the Oxford Vaccine Group (OVG) was used as a template for the ComFluCOV trial database, which was built in REDCap (https://www.vanderbilt.edu/). The dataset and associated database specifications for ComCOV were updated by a trial manager and reviewed by the trial statisticians to ensure all data required to report the primary and secondary outcomes for ComFluCOV were collected in the format suitable for analyses. The database was then tested by a trial manager who followed the usual testing procedure implemented for BTC trials; all possible combinations of test data were entered to check the functionality worked as anticipated (e.g. if a value was entered outside of a pre-specified range, a warning message should appear). Any updates required were made, and the database specifications were signed off. Data collection was fully electronic. Participants completed electronic symptom diaries (e-diaries) for seven consecutive days following vaccinations at visits one and two and any time they experienced an unsolicited or medically attended event thereafter. If no e-diary was completed after day 7, it was assumed no unsolicited or medically attended events occurred and participants were asked by site staff to confirm the information at the following study visit. E-diaries were sent to participants to complete via an email link which took them to a REDCap survey. The link was initially open for 24 h, but part way through recruitment, this was extended to 48 h to allow participants who missed the 24-h window to access the link but were able to remember and report symptoms from the previous day. Only the site staff and the BTC team had access to the trial database which was stored on a secure server hosted by the University of Bristol.

### Monitoring, analysis, and reporting

**Fig. 3:**
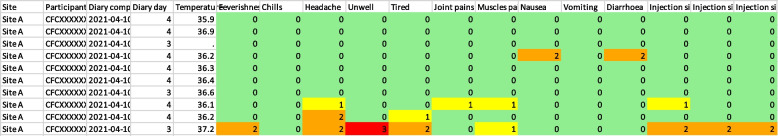
Screenshot of report sent to sites highlighting adverse events

Data reports were prepared for the central trial team at the BTC, sites, and independent oversight committees. Reports for the trial team and sites were automated and produced 7 days a week. Two types of automated reports were produced: (i) a report of data collected at study visits was produced following each clinic session and morning and evening as well as ad hoc when requested by trial managers, and (ii) a report of participant completed e-diaries was produced every morning throughout the trial until the last participant completed their final e-diary entry. The first automated report included summaries of recruitment, protocol adherence, sample collection details, and data quality. Also included were baseline demographics of randomised participants, any duplicate participant identification numbers (as REDCap allows the same identification number used for more than one participant), and participant identification numbers of pregnant participants randomised to ensure they were followed up appropriately (i.e. until delivery to screen for adverse pregnancy outcomes). Separate reports were produced for each clinic session at each site. Reports were reviewed by a trial manager shortly after they were available, and any queries identified in the reports were entered into a central query log and sent to the trial sites, to aid sites in addressing queries in real time. Reports were reproduced on successive days until all queries identified from the clinic session had been resolved which was confirmed by a trial manager on the central query log. The second report listed the trial identification numbers of any participants who had not yet completed their e-diary and highlighted any adverse events the participant had reported using colour coded Excel spreadsheets, where certain colours highlighted the events which required additional attention due to their severity (Fig. 3). These reports were sent each morning via email by the trial managers to clinical teams at trial sites who had been delegated the task of safety oversight. Systematic reminders were also set-up within the database to ensure participants who had not completed their e-diary were reminded to do so via email.

The statistical analysis plan (SAP) was written by the trial statisticians using a template developed at the BTC for all trials. The SAP described in detail all derivations that were essential for analyses and the statistical approaches that were to be followed for all outcomes/analyses, including any key assumptions that would be made. The SAP did not include any detailed analysis code as this is not usual practice for the BTC. After review, it was signed off by the chief investigator and the senior statistician. An interim analysis for the DHSC was completed after the SAP had been signed off. This report contained an analysis of the primary outcome as well as summaries of recruitment to date, protocol compliance, safety, and completeness of clinic data and e-diaries. This interim analysis was requested by the UK government to inform discussions regarding the autumn (September–November) vaccine policy. No stopping rules for efficacy or harm were defined as it was always intended the trial would continue unless the independent data monitoring and safety committee (DMSC) recommended stopping due to harm.

Final data cleaning began once the final participant randomised had completed their follow-up. Individual reports listing any unresolved queries that had not been picked up in the daily monitoring (i.e. missing data fields required for each participant as well as any queries on the data entered) were sent to sites by the trial management team. All queries were collated by the trial managers in the central query log and followed up to resolution, with sites updating the trial database where necessary. Excel spreadsheets were used to collect any additional and/or updated data that was reported outside of the daily participant e-diaries, as the trial database functionality did not permit diary entries to be updated once completed and saved by the participant. The spreadsheet included a column for each data item present in the e-diary and for any associated comments as well as the participant identification number and date the data related to which allowed the data to be formatted and merged with the data extracted from the database. These data included diary entries collected by the local sites and passed on to the trial managers if the participant had missed the window to complete the e-diary using the link provided by email, updates to body temperature or severity of reactions, or details of when a participant had completed their e-diary using the wrong e-diary link (e.g. reporting the symptoms a day late). Data for immunogenicity outcomes was not entered into the database but was provided directly by the laboratories completing the analytical work.

Once data cleaning was complete, the database was locked, and final analyses began. Both trial statisticians completed the analyses of all outcomes independently in parallel, following the methods pre-specified in the SAP. The derivations and results of all analyses were then compared and any discrepancies found were discussed and resolved. Results were presented internally to the trial team and reports were prepared for the DHSC, oversight committees, and peer-reviewed publication.

## Results

### Study set-up

Study set up began in early March 2021 and was completed less than a month later. Pre-screening tools to calculate age and eligibility windows were developed by one of the trial statisticians (RTh) within 2 weeks and were ready for the first site greenlight, aiding those completing telephone screening of potential participants. The intention was to blind both trial statisticians to the allocation; however, the blinding of study statisticians was not implemented as planned due the time constraints and the need for the trial statisticians to monitor safety aspects of the intervention including intervention-specific batch numbers. The chief investigator and senior statistician remained blinded. Recruitment to four cohorts (two COVID-19 vaccines and two influenza vaccines) began in April 2021. In the week before opening to recruitment, the DHSC requested the trial be adapted to include a third influenza vaccine. Recruitment to the four cohorts continued whilst the adaptations were made. Ninety-one participants were randomised on the first day of recruitment, and a total of 492 participants had been randomised when the adaptation was complete and sites were ready to start recruiting to the fifth and six cohorts. The adaptation required an update to the randomisation scheme to incorporate the two additional cohorts. The statistician who prepared the initial randomisation scheme provided the additional allocations to the database manager, who uploaded the data into the trial database. No other amendments to the database or testing of the database functionality was required. Meetings with a senior statistician from OVG, who worked on other ComCOV studies, were held ahead of receiving any laboratory data from analyses of blood samples, to discuss the format in which the immunogenicity data would be received and to ensure that the same statistical approaches as those used in other ComCOV studies were used for consistency.

### Monitoring

Clinics took place on weekdays and weekends and the trial statisticians provided out of hours and weekend support. Our risk-proportionate approach to monitoring determined that the coding of the specific reports was undertaken by one statistician with oversight from the other. Five-hundred and thirty-seven reports summarising data collected at clinics at each site were produced between 1 April 2021 and 19 July 2021. In total, 1874 data queries across the 12 recruiting sites were identified, logged in a central query log maintained by the trial managers, sent to sites, and resolved as a result of these reports (Table 1). The most frequently appearing queries were staff signing off forms when they were not delegated to do so and were rectified by appropriately delegated staff reviewing and signing off, and missing sample processing data, which was often rectified as soon as the laboratories processed the samples. A total of 695 reports identifying missing e-diaries and adverse events were produced between 2 April 2021 and 15 July 2021. These reports were particularly important as the e-diaries provided primary outcome and adverse event data, and if missing diaries were identified early enough, the information could still be collected outside of the e-diary system by the local site team from the participant. This extensive monitoring of data resulted in high data completeness rates; the primary outcome (any solicited systemic reaction in the 7 days following vaccination) was complete for 651/679 (96%) participants following visit 1 and 581/679 (86%) following visit 2. The secondary outcome of any local adverse reaction was complete for 665/679 (98%) participants following visit 1 and 619/679 (91%) following visit 2. Of those with missing data, eight formally withdrew from the trial. The code to produce the reports was written in Stata version 17. The *putdocx* suite of Stata commands were used to create Office Open XML (.docx) documents. Early in the study, the commands ran successfully, and reports were produced automatically using a shell script. Once the dataset reached a few thousands of observations, the code took several hours to run and would frequently crash as the *putexcel* command interfered with the file being written in the storage directory. Thereafter, the reports were generated individually and appended to the results of the previous day.

**Table 1:** Number of data queries identified from clinic reports by site

Query type	Site 1	Site 2	Site 3	Site 4	Site 5	Site 6	Site 7	Site 8	Site 9	Site 10	Site 11	Site 12
**Total queries**	**274**	**163**	**194**	**150**	**107**	**461**	**351**	**8**	**79**	**40**	**17**	**30**
Adverse events	25	22	20	9	0	27	4	2	5	0	0	4
Batch numbers	4	1	10	0	0	11	0	0	0	0	0	0
Consent forms	2	2	3	4	0	8	0	1	0	3	0	4
Data entry	60	86	20	34	29	56	40	2	3	6	8	20
Eligibility	8	1	0	0	0	0	0	0	1	1	0	0
GP letter	2	0	0	81	0	1	19	0	11	0	0	0
Intervention	1	5	0	0	0	14	3	0	0	1	3	1
Missing data	35	31	77	9	15	48	9	2	12	5	2	1
Out of window visit	0	0	2	0	0	1	0	0	0	0	0	0
Pregnant participant	0	1	0	1	0	0	0	0	0	0	0	0
Sample data	137	14	62	12	63	296	276	1	47	24	4	0

### Analyses and reporting

The first report to DMSC and trial steering committee (TSC) was produced one month after recruitment started in April 2021 by which point 197 participants had been randomised. The statistical analysis plan was written and signed off in May 2021. A report for the DHSC containing interim primary outcome analyses of validated data was completed 6 days later, 7 days after 473 participants had complete data necessary to derive the primary outcome. At this time, recruitment to the first four cohorts was complete, and these analyses were used to inform the DHSC policy. The oversight committees received a second report summarising progress on the study in June 2021.

Recruitment of 679 participants was complete in 8 weeks. Final data cleaning began shortly after the final participant completed follow-up. Data cleaning was mainly focussed on data collected outside of the trial database and note to files (extra information collected that did not fit anywhere else in the database) as the majority of clinic data had already been cleaned by regular monitoring throughout the study. The final data lock took place 6 weeks after the final participant completed followed up.

Final analyses took place over several weeks. We identified that the analysis presented the highest risk from a statistical perspective considering the model assumptions, potential for bias and statistical packages that can be used to conduct the analyses. The risk we assigned to each aspect of the trial is shown in Table 2. Given the complexity, data management steps, and impact of an error, the risk-proportionate approach for the analysis determined that each statistician should perform their analyses separately, thereby double-coding the outcome derivations and modelling. Both statisticians performed all analyses in Stata. The highest risk arises from the intentional or unintentional use of a specific package, interpretation of the model assumptions checks, potential mistakes, and other choices a statistician can make whilst conducting the analyses. When the derivations and results were compared, minor differences in the coding of additional data collected outside of the database were identified. Discrepancies were found in the derivation of the primary outcome for approximately 0.02% (~12 discrepancies) of diary days completed and in the derivation for one of the sensitivity analyses due to a misspecification in the imputation model. Differences were resolved; derivations were re-run and confirmed to match. All statistical modelling approaches were consistent, and definitive analyses were completed 3 weeks after data lock. Results were sent to the DHSC and policy stakeholders, with a pre-print publication within 14 days of the results being made available to the study team. The results of the trial were published on the Lancet website in November 2021 and were published in the Lancet journal in December 2021, 10 months after first discussions about the trial.

**Table 2:** Aspect of study and level of risk assigned

Aspect of study	Level of risk	Action
Screening tools for sites	Low	One study statistician created the tools
Reports of clinic data	Low	One study statistician produced the report
Reports of e-diaries	Low	One study statistician produced the report
Randomisation scheme	Medium	Two statisticians (one study statistician blinded when the randomisation scheme was produced created the test randomisation scheme, and one unblinded independent statistician produced the final randomisation scheme)
DMSC/TSC reports	Medium	Both study statisticians worked together to create the report
Preliminary report for DHSC	High	Both study statisticians created all tables and figures for report
Derivation of outcomes	High	Both study statisticians derived all outcomes independently and compared derivations
Modelling of outcomes	High	Both study statisticians analysed all outcomes independently and compared results
Final report for DHSC	High	Both study statisticians created all tables and figures for report

## Discussion

The methodologies the BTC statistics team implemented in ComFluCOV helped the study to be delivered to inform the autumn 2021 vaccination policy. Results of the trial were delivered on time, and the implementation of coadministration of COVID-19 and influenza vaccines is now underway in the UK and overseas, where feasible.

We overcame many challenges during the delivery of the trial. ComFluCOV was the first vaccine trial for the BTC and therefore was a new clinical field. Meetings with research teams who were more familiar with the type of immunogenicity data collected in vaccine trials, held prior to the ComFluCOV analysis stage, allowed the ComFluCOV statisticians to gain an understanding of the format of the data that would be received as well as important steps required during analysis. This saved time and resources once the final data were received and allowed the analyses to be undertaken immediately using the agreed statistical approaches. The BTC usually builds trial databases using an in-house system; however, REDCap was used for speed in this case. This meant learning about the features and processes used in REDCap in order to develop database specifications, export, and format the datasets and view data queries in just a few weeks, which was a steep learning curve. The team incorrectly assumed that the key features of BTC in-house database systems, such as ensuring all participant identification numbers are unique, would apply to the REDCap database; however, this was not a standard feature in REDCap and required additional checks. Lastly, our most challenging issue was reporting timescales were much tighter than for other trials. Study data had to be analysed in weeks rather than months.

Several features were key to our success. We had daily contact with the core trial team (senior oversight, trial managers and database managers) which meant we were up to date with study progress and aware of upcoming developments. The decision to adapt the ComCOV database provided by the OVG for ComFluCOV saved approximately 2 to 3 weeks in development time compared to starting from scratch. The trial made other time-efficiency and pragmatic decisions such as deciding not to blind both trial statisticians before any data collection to alleviate pressure on an already complex database system and using readily available software such as Excel for a specific purpose even if not usually the preferred solution. We invested time at the start of the trial to automate the daily data monitoring reports. This provided consistency and allowed them to be produced quickly whenever they were required, which saved us valuable time given the vast number of reports produced throughout the trial. The real-time monitoring of trial data and feedback to trial teams streamlined the data cleaning process and resulted in high quality data. The primary and secondary safety outcomes had over 85% data completeness, which, given these outcomes were participant reported, is high. The two trial statisticians already worked closely together as part of the wider BTC statistics team who all work in a similar way following similar structures and methodologies. This resulted in a seamless transition to working directly together on this trial. The processes and techniques used in ComFluCOV have been further adapted and are being implemented as standard practice for some other trials that require frequent monitoring. Methods that failed during the course of the trial (e.g. the program crashes) have been improved, and the methodology is being used in other studies. Finally, having two trial statisticians working together on all aspects of the trial allowed reports to be prepared and checked in a timely manner, minimised the risk that coding errors would go undetected, data were interpreted correctly, deadlines were met, and the whole process was quality assured.

The trial had several limitations from a statistical perspective. Firstly, it was not ideal practice to add new research cohorts once recruitment had already started. However, this was essential as we needed to respond to the request from the UK DHSC and the needs of the pandemic as quickly as possible, and this adaptation was necessary for the study to address the research question as fully as possible. Had time and resources allowed, we would have assigned another statistician to the team to check batch numbers and other data that unblinded the trial statisticians. However, the quality checks implemented minimised bias that could have occurred due to the statisticians being aware of the trial allocations. Similarly, if more time had been available for developing the trial database, we would have ensured that it was not possible to add duplicate participant identification numbers. This has been implemented for a new vaccine trial the BTC are running which also uses REDCap for the trial database. In this trial, we have also added an e-diary review form to the database to make it easier to track which adverse events reported in the participant e-diaries have been reviewed by a clinician and the outcome of the review. This process was organised manually in a spreadsheet in ComFluCOV. Ideally, we would have written the SAP earlier in the trial, but time constraints did not permit this. However, the statistical approaches to analyses were outlined in the protocol; the SAP expanded on the methods outlined at the outset, and no changes were made to what was planned, so we do not believe any additional bias arose due to the SAP being written after recruitment had started. The sample size did not account for dropout as given the nature of the primary outcome we did not anticipate dropout would be a problem. We had complete primary outcome data for 96% of randomised participants and partially complete primary outcome data for the remaining 4%. Sensitivity analyses to account for missing data were performed and were consistent with the primary analysis. The proportion of missing data in this trial could be used to inform future sample size calculations using the same primary outcome in order to account for missing data. Similarly, although we did not account for clustering in the sample size calculation, analyses adjusted for the clustering within centre where possible. Finally, we could have increased the independence of analyses by having each study statistician completing analyses in a different statistical software (e.g. one using Stata and the other R); however, both statisticians were more proficient in Stata, and we believed the increased risk of errors arising from using a less familiar software package under short timescales outweighed the benefit of using two different software packages to complete the analyses.

## Conclusion

The ComFluCOV trial results were reported within 6 months of conception of the trial. Having two statisticians working together, deriving and analysing, all outcomes provided a quality assurance process that enabled all reporting and analyses to be completed both efficiently and accurately. The risk-based approach allowed limited resources to be appropriately allocated to each stage of the trial. Lessons learnt from this experience are being applied to other studies in the BTC and could be considered to increase efficiency and give a quality assurance process in other studies.

## Data Availability

Not applicable.

## Abbreviations

BTC: Bristol Trials Centre
COVID-19: Coronavirus disease 2019
DHSC: Department Health and Social Care
DMSC: Data monitoring and safety committee
OVG: Oxford Vaccine Group
RCT: Randomised controlled trial
SAP: Statistical analysis plan
TSC: Trial steering committee
